# Bird-window collisions: different fall and winter risk and protective factors

**DOI:** 10.7717/peerj.9401

**Published:** 2020-06-19

**Authors:** Barbara B. Brown, Lia Hunter, Sabrina Santos

**Affiliations:** 1 Family & Consumer Studies, University of Utah, Salt Lake City, UT, United States of America; 2 Environmental and Sustainability Studies, University of Utah, Salt Lake City, UT, United States of America

**Keywords:** Bird, Window collision/strikes, Prevention, Reflective windows, Fruiting trees, Bird-friendly window, Fritted window, Pear trees, Cedar Waxwings, Seasons

## Abstract

**Background:**

To reduce bird fatalities from millions of window collisions each year in North America, it is important to understand how design and landscape elements relate to collision risk. The current study extends prior research that found that buildings near ornamental pear trees (*Prunus calleryana*) and buildings with mirrored windows significantly increased odds of collisions among eight buildings on the University of Utah campus in winter. The previous study found bird-friendly glass was not related to collision risk, although only one fatality occurred at two buildings with ORNILUX® ultraviolet (UV) or fritted windows. We reasoned that extending data collection to include fall might provide a better test of efficacy. We tested the following three hypotheses: (1) Buildings with mirrored windows would experience more collisions, replicating the original study; (2) the addition of fall migration data would reveal fewer collisions at the buildings with bird-friendly windows; (3) the danger of pear tree proximity would be heightened in winter, when fruit is ripe enough to appeal to frugivores, especially the Cedar Waxwings (*Bombycilla cedrorum*) that frequent these trees.

**Methods:**

Trained observers monitored buildings three times per week in Fall (September 12 to October 27, 2019) and Winter (October 29, 2019 to January 24, 2020). Collisions were photographed and documented in the iNaturalist University of Utah Bird Window Collision Project.

**Results:**

There were 39 total collisions, from 0 to 14 per building**.**Using generalized estimating equations, buildings near pear trees had 3.33-fold increased odds, mirrored windows had 5.92-fold increased odds, and bird-friendly windows had an 84% lower odds (Odds ratio = 0.16) of bird window collisions when analyzed separately; all were statistically significant (*p* < 0.01). A test of all possible combinations of risk and protective factors revealed that the best fit model included pear trees (odds = 2.31) and mirrored windows (odds = 2.33). A separate analysis tested the pear tree by season interaction model; it yielded the deadliest combination, with 40-fold increased odds for buildings near pear trees in winter season.

**Discussion:**

This research provides the first peer-reviewed evidence found for the efficacy of bird-friendly fritted windows and ORNILUX ® UV windows in buildings. In addition, it replicated a study that established the dangers of mirrored windows and fruiting pear trees near buildings. These risks were especially dangerous to Cedar Waxwings, who constituted 62.2% of the identifiable window collision victims. This research highlights how building risks depend on window design, landscape choices, species, and season. If replicated, analyses of risk factors can help identify buildings that require mitigation to make existing windows less deadly. Results also support the installation of bird-friendly glass in new or renovated buildings to reduce fatalities.

## Introduction

In the United States, up to approximately one billion birds die each year from colliding with windows on buildings (Loss et al., 2014). These fatalities could be reduced by using mitigation techniques, such as the application of markers on window exteriors that are spaced at most five cm apart horizontally by 10 cm apart vertically—the 2″ by 4″ rule (Klem, 2008; Klem, 2009)—to make them visible to birds (see also Rössler, Nemeth & Bruckner, 2015; Sheppard & Phillips, 2015). More permanent potential solutions involve installations of bird-friendly glass that creates visibility for birds through frits, etching, or ultraviolet patterns (Klem, 2009; Sheppard, 2019). However, much of the research demonstrating the efficacy of window protections has involved experimental tests of windows that are not attached to buildings. These experimental tests provide valuable insight into conditions that can reduce the deadliness of windows. However, each building has a “mortality signature,” based on features of the building and surrounding habitat (Hager et al., 2013). Therefore, field work is needed to identify how collision risk and prevention factors perform around existing buildings. We focused on risk factors identified in our prior winter-season study that found high risks for mirrored windows and nearby fruiting pear trees (*Prunus calleryana*), where Cedar Waxwings (*Bombycilla cedrorum*) were especially likely to feed on ornamental pears (Brown et al., 2019). We extended our prior research by including both fall and winter season data. We expected that buildings with pear tree proximity would have more winter collisions among frugivores, and that both seasons combined would show mirrored windows have higher risks while permanent window protections have lower collision risks.

### Bird-unfriendly windows

The dangers posed to birds by transparent, reflective, or mirrored glass have been demonstrated in both experimental tests and correlational field studies. Birds avoid certain window treatments when treated and control windows are hung at the end of flight tunnels (Klem, 2009; Klem & Saenger, 2013; Rössler, Nemeth & Bruckner, 2015) or in fields close to a wooded area (Klem, 1989; Klem, 1990; Klem, 2009). For existing buildings, the presence or extent of windows has correlated with greater bird mortality (Borden et al., 2010; Cusa, Jackson & Mesure, 2015; Hager et al., 2013; Klem, 1990; Klem, 2009). As Klem noted, “birds behave as if windows are invisible” (Klem, 2009). Mirrored glass has rarely been singled out for evaluation against other window designs, although past research has noted that mirrored windows pose high collision risks (Evans Ogden, 2002; O’Connell, 2001; Sheppard & Phillips, 2015). We examined whether a mirrored glass façade that partially covered one of eight monitored buildings had higher collision risks, which would replicate research from Brown et al. (2019).

### Bird-friendly windows

Given the dangers of traditional reflective and mirrored windows, some buildings now have bird-friendly windows, defined as windows provided with permanent features visible to birds that deter collisions. These include fritted windows with small ceramic frits fused into the glass to reduce light and solar heat penetration (Wilson & Elstner, 2018). As a secondary benefit, these frits also are more visible to birds; for example in outdoor cage tests, birds avoided fritted windows in 62 of 70 trials (Klem, 2009). However, the frits are visible to humans, which may limit the acceptability of this solution.

Other permanent bird-friendly windows include embedded ultraviolet patterns, which are very unobtrusive to humans, such as the Mikado pick-up stick pattern from ORNILUX^®^. In experimental tests these windows reduced collisions when the interior was dark (Klem & Saenger, 2013). However, bird species may vary in their abilities to detect these patterns (Håstad & Ödeen, 2014) and ultraviolet light is less available on overcast days or early mornings (Sheppard, 2019).

### Trees create collision risks

Among the habitat factors that create window collision risks, trees and shrubs close to buildings or reflected in windows have also been found to increase collision risks (Borden et al., 2010; Gelb & Delacretaz, 2006; Klem et al., 2009; Kummer, Bayne & Machtans, 2016b). Although some past research focused on risks associated with particular trees, such as London plane trees (*Platanus x acerifolia*) (Gelb & Delacretaz, 2006), most studies do not specify particular species as creating risks. Pear trees became an initial focus for our study given early observations of the presence of Cedar Waxwings, who are especially dependent on fruit as a food source in winter (Witmer, 1996; Witmer, Mountjoy & Elliot, 2014). Because fruiting ornamental pear trees provide Cedar Waxwings with essential winter food and attract them to trees near buildings at the University of Utah, we focus on pear trees as a key risk factor.

### Fall/winter replication rationale

In Brown et al. (2019), we examined how mirrored windows, pear trees near buildings, and bird-friendly buildings related to collision risks among eight buildings during winter season. We found that, when examined in single-variable models, pear trees near windows created an 8.5-fold increased odds of collisions and that mirrored windows created a 5-fold increased odds of collisions (Brown et al., 2019). Two buildings had bird-friendly windows, one with fritted and one with ORNILUX^®^, and only one window collision was recorded across both buildings (a feather pile detected at the building with fritted windows). However, given that only two of eight buildings in that study had frequent window collisions in winter, the differences were statistically non-significant between the two buildings with bird-friendly windows and the other six buildings. Some of the six buildings without bird-friendly windows were known, from ad-hoc reports, to have experienced collisions in non-winter seasons. Research suggests that collisions are more numerous in migration seasons (Borden et al., 2010; Hager et al., 2013; Kahle, Flannery & Dumbacher, 2016; Schneider et al., 2018), especially fall (Loss, Will & Marra, 2015). We reasoned that an extended observation period, including fall and winter, could provide a more robust test of the efficacy of bird-friendly windows compared to other windows.

By extending the observation times to fall and winter seasons, we were able to address several research aims. First, we tested whether buildings with mirrored windows experienced more collisions, a finding that would replicate the original study (Brown et al., 2019). Second, we expected that the addition of fall migration data would show that the buildings with bird-friendly windows would have fewer collisions than other buildings. Third, we expected that the danger of pear tree proximity would only emerge as a significant factor in winter (a pear tree by season interaction), when birds eat the fruit.

## Methods

### Study site

The University of Utah is located along semiarid foothills of the northeastern edge of Salt Lake City, Utah, at the foot of the Wasatch mountains (interior US Environmental Protection Agency Ecoregion 19, Environmental Protection Agency, 2018). Its grounds are designated as a state arboretum. Therefore, it has a number of native species, such as Blue Spruce (*Picea pungens*), white fir (*Abies concolor*), and Gambel oak (*Quercus gambellii*), as well as introduced species, such as English hawthorn **(***Crataegus laevigata*). Fruiting trees such as ornamental pear trees (*Prunus calleryana*) and flowering crabapples (multiple *Malus* species) are common on campus.

### Building risk and protective factors

We selected buildings and operationalized their three risk and protective factors as previously described in Brown et al. (2019). Table 1 shows the eight buildings, their collision totals, and their risk and protective factors, along with window areas for monitored facades. Three buildings (1, 2, and 4) had mature fruiting pear trees within 4.9 m of the building façades (Brown et al., 2019). Building 1 had the risk factors of a mirrored façade on a 1980s extension to a 1901 building. It had 119 m^2^ of mirrored surface, of which 30% or approximately 36 m^2^ were protected with Feather Friendly^®^ Symmetry mitigation treatment. In addition, it had 114 m ^2^of traditional reflective windows on the other facades. The 1980s-era mirrored glass was more than just reflective; it included large mirrored sheets that reflected images even as natural light was dimming and regardless of the amount of sunshine. The mirrored sheets spanned across structures in the building, such as floors, and these structures were invisible to a person standing outside the building. The mirrors blocked so much light coming into the building that even on a bright day the building has overhead lights on inside to illuminate the hallway behind the mirror.

**Table 1:** Total risk and protective factors and collisions by building and season.

		**Risk and protective factors present for each building**			**Collisions by season and building**		
**Building**	**Window area, m**^**2**^	**Pear trees**	**Mirrored windows**	**Bird-friendly glass**	**Fall**	**Winter**	**Total**
1. AEB	144	1	1	0	0	14	14
2. JTB	431	1	0	0	1	10	11
3. CSC	660	0	0	0	0	1	1
4. LCB	788	1	0	0	1	0	1
5. MEB	3,342	0	0	0	4	1	5
6. WEB	1,219	0	0	0	4	1	5
7. Law	1,860	0	0	1	2	0	2
8. GC	1,002	0	0	1	0	0	0
Total					12	27	39

**Notes.**

Building 1 also had 119 m^2^ of mirrored windows. Data were collected in fall (September 12–October 27, 2019) and winter (October 29, 2019–January 24, 2020) at the University of Utah, Salt Lake City Utah, USA. Monitoring included all four sides (for buildings 1, 2, 4, 5, and 6) and particular sides (for 3, 7, and 8).

**Figure 1:**
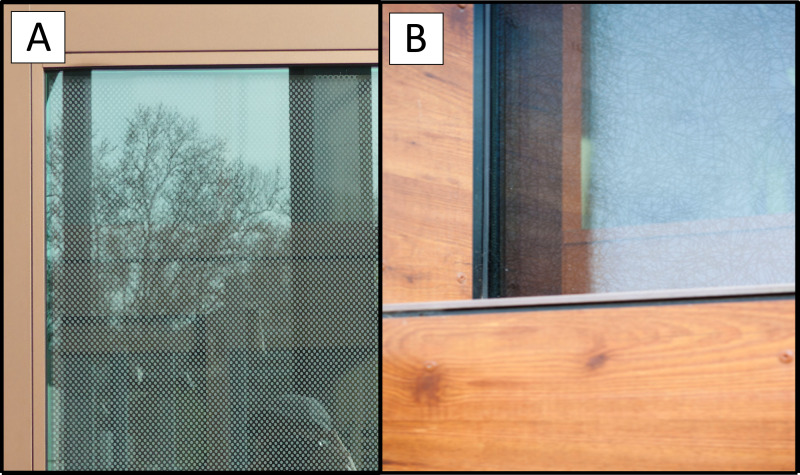
Bird-friendly windows including (A) fritted window and (B) Arnold Glas/ORNILUX bird protection glass. Photo credits: (A) Bill Hanewinkel and (B) Lisa Schon (Arnold Glas Corp.).

Finally, two buildings had bird-friendly glass, as shown in Fig. 1. Building 8 had windows with grey circular frits, approximately 3.2 mm in diameter, spaced at approximately 4 dots per 25 mm, arranged in staggered rows, covering approximately 40% of the surface. The ORNILUX^®^ Bird Protection Glass Mikado pattern looks like faint images of pick-up sticks criss-crossing in the glass, which had Polaris low-e coating (Lisa Welch, personal communication, Arnold Glas). The ultraviolet coating is designed to be visible to birds without obstructing human views. The three other buildings were selected because they had anecdotal reports of collisions, large amounts of traditional reflective glass, or proximity to many tree species.

### Collision monitoring

#### Collision definition

Collisions were defined as any stunned birds, bird carcasses, or feather piles (>12 feathers within 0.09 m^2^) found under windows and extending 3 m from the designated building sides. This excluded any carcasses found under walls without windows, or situated under trees in front of walls or along hand rails or other structures around buildings but not under windows. Such carcasses were likely eaten by predators perched on rails or in trees. We should note that a Mourning Dove carcass looked as though it had been cleaned by a predator, who might have been perched on the building overhang for Building 7. The bird might have hit a window, then been scavenged, or a predator might have killed it elsewhere but perched on the building to eat it. The carcass met our definition of a window collision, so we kept it in the data set.

#### Data documentation

Observers verified their building observations by taking time-stamped cellphone photos of each building they observed, with the name having the format: year, month, day, hour, and minute. In addition, rater initials were added to the end of any photo uploaded by any observer other than the first author. We uploaded photos to a shared drive that included one folder per building and a separate folder documenting any carcasses or stunned birds. The photographs of carcasses and stunned birds, where possible, can be viewed on the iNaturalist project page (see https://www.inaturalist.org/projects/university-of-utah-bird-window-collision-project).

#### Observer training and procedures

**Figure 2:**
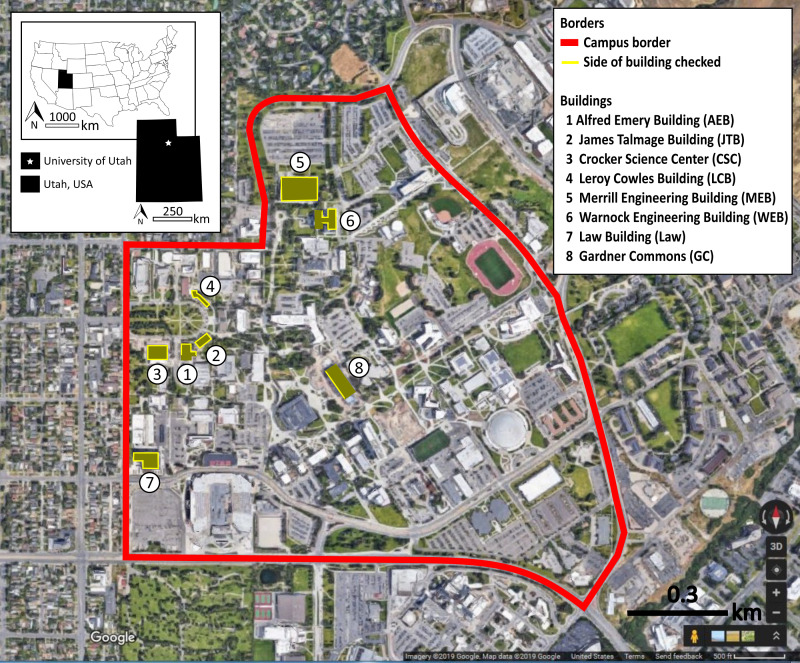
Map of the eight monitored buildings. The solid lines around buildings indicate which sides were monitored at the University of Utah, Salt Lake City, Utah, USA. Map data ©2019 Google.

Observers received on-site training and collected inter-rater reliability data. Four observers collected most observations, supplemented in January by five volunteers from a conservation biology class. Training included walking along and, for the main observers, detection of carcasses placed below windows as training exercises, and instruction to alternate clockwise and counter-clockwise directions around buildings across monitoring days. In some cases, consistent with past research (Borden et al., 2010), the full building circumference was not monitored. Building 8 had fritted windows only on the west façade, so we monitored that façade only. Part of Building 1 was obstructed by a fenced in playground and part of Building 6 was inaccessible due to a rocky hillside. Figure 2 shows the monitored buildings, including designation of monitored facades, which were identical to Brown et al. (2019). Observers were instructed to double-bag carcasses or feather piles for delivery to an ornithology lab, which had an appropriate salvage license (United State Fish and Wildlife Service permit # MB836059-0, and Utah Division of Wildlife Resources Certificate of Registration # 5COLL3669) for research and disposal (Hager & Cosentino, 2014).

#### Interrater reliability data

Although schedules permitted only one observer in most cases, we assessed the interrater reliability of pairs of observers for 224 building observations. This involved two observers evaluating the same building at the same time, going in opposite directions around a building. They were instructed that if they found a bird during a reliability check they should take a quick picture of it immediately, so as not to delay completion of the route and thereby forewarn the other rater of the presence of a carcass. After route completion, observers compared their collision counts, showing each other any photos they took of carcasses. Only after completion of their building circuit did the observers take the time for close-up photos needed to identify the bird and distant photos needed to identify the building location.

#### Data collection dates and times

Fall monitoring occurred September 12 to October 27, 2019. Winter observations occurred October 29, 2019 to January 24, 2020, similar to the November through January time from the prior study (Brown et al., 2019). The winter end date occurred before a string of 10 straight days of no collisions; at that point the pear trees had been stripped of almost all fruit. Because a snowstorm occurred toward the end of the 10-day period, we extended the observations into mid-February when snow receded, to assure that no carcasses buried under the snow had been missed; we did not detect any buried carcasses by doing this extra monitoring. We monitored three times a week, which is an interval that should allow us to detect most carcasses, based on studies that determined predators would take about that long or longer to remove carcasses (Hager, Cosentino & McKay, 2012; Hager & Craig, 2014; Kummer et al., 2016a; Riding & Loss, 2018). Observation times during the day varied according to observer availability. Study procedures received permission for data collection from the University of Utah institutional review board (IRB_00117279).

#### Outreach

**Figure 3:**
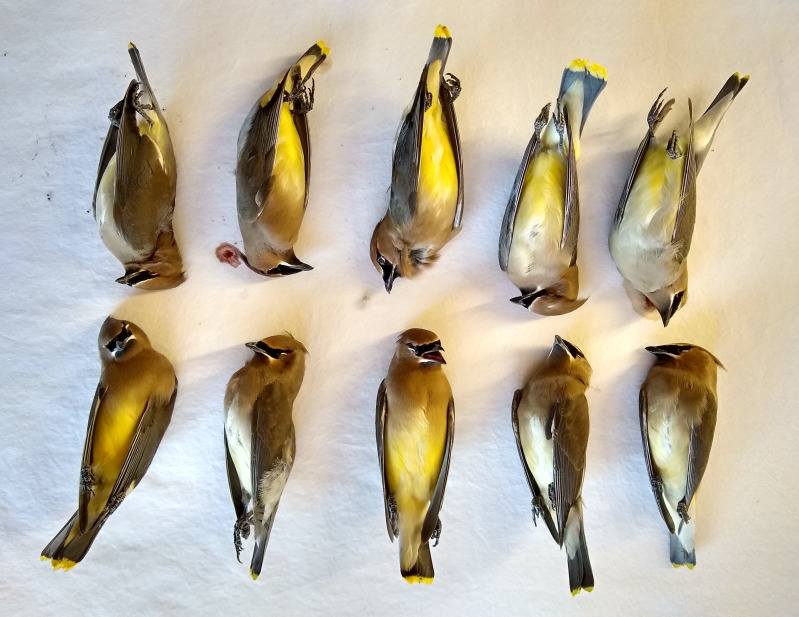
Ten Cedar Waxwings dead from collisions on two adjacent buildings. Photo credit: Bill Hanewinkel.

To assure that we did not miss any hotspots of bird window collisions, we installed posters around campus with our contact information and published a newspaper editorial part way through the study. These outreach measures allowed us to share our iNaturalist site with campus users and to keep us apprised of any other problem areas on campus and provide public documentation of the study, as recommended by other researchers who focus on window collisions (Winton, Ocampo-Peñuela & Cagle, 2018). We also posted to iNaturalist and the local paper one striking photo of the results from the deadliest day of observations in order to sustain interest and concern for mitigation solutions (Fig. 3).

### Data analysis

To assess interrater reliability we used the intraclass correlation coefficient (ICC), in which a score of 1.0, on a 0 to 1.0 scale, indicates perfect agreement (Shrout & Fleiss, 1979). The ICC scores within 95% confidence intervals are interpreted as evidence of good reliability if values are between 0.75 and 0.90 and excellent reliability if values are above 0.90 (Koo & Li, 2016). We used a one-way random effects test to derive the ICC, which is appropriate when different observers contribute to reliability observations. We selected the value suitable for reliance on a single rater’s score rather instead of an average score across observers (Koo & Li, 2016). All analyses used IBM SPSS Statistics 25 software (IBM, 2017, Armonk, NY, USA).

To assess the effects of risk and protective factors, we used the daily count of bird window collisions for each of the eight buildings. We employed Generalized Estimating Equations (GEE), which are useful for analyses of correlated count data across repeated measures, such as our repeated assessments of each building (Norusis, 2008). Our model used a Poisson distribution, log-link function, and significance levels of *p* < 0.05 (Ma, Mazumdar & Memtsoudis, 2012). After preliminary testing of an autoregressive structure across repeated weeks, we selected an independent correlation structure instead, given slightly better model fit results. The fit statistic used to compare models was a quasi-likelihood under independence model criterion (QICC); smaller scores indicate better fit (Norusis, 2008). We entered the three risk and protective factors as dummy variables in an analysis that included both fall and winter data. Tested models replicated the past analyses of Brown et al. (2019). These included a test of all three risk and protective factors, followed by each factor tested alone, followed by the best fit model from all 1-, 2-, and 3-way tests. To evaluate the expectation that pear tree proximity was especially deadly for birds in winter, in a new analysis we added a main effect for season (0 = fall and 1 = winter) and the key season by pear tree interaction term. If our hypothesis is correct, there should be more collisions counted in the winter season for buildings near pear trees than for other combinations of seasons and pear tree proximity (i.e., fall/pear trees, fall/no pear trees, and winter/no pear trees). We also presented simple descriptions of window areas and counts of collisions by buildings and season in Table 1.

## Results

Reliability checks showed that observers had good to excellent agreement, with an ICC of 0.92 (95% confidence interval of 0.89 to 0.94).

There were 12 collisions in fall and 27 in winter for a total of 39 (see Table 1). Buildings 1 and 2, which were close to pear trees, including the one with the mirrored façade, showed the highest numbers of window collisions. The other six buildings had between 0 and 5 window collisions. Buildings 1 and 2 had 14 and 11 collisions, respectively, in the current study and had 10 and 9 collisions in the Brown et al. (2019) study, suggesting stability of risk. As shown in Table 2, all but one of the collisions in the current study occurred in winter and all but 2 of the 25 collisions at buildings 1 and 2 were Cedar Waxwings. In sum, 62.2% of the 37 identifiable birds were Cedar Waxwings. There were only 3 winter collisions at any of the other six buildings.

**Table 2:** Number of window collisions per species by building and fall (F) and winter (W) season.

**Common name**	**Scientific name**	**1**	**2**	**3**	**4**	**5**	**6**	**7**	**8**	**Totals**
Cedar Waxwing	*Bombycilla cedrorum*	14-W	9-W	0	0	0	0	0	0	23
Lincoln’s Sparrow	*Melospiza lincolnii*	0	0	0	0	0	1-F	1-F	0	2
Yellow-rumped Warbler	*Setophaga coronata*	0	0	0	0	0	1-F, 1-W	0	0	2
Mourning Dove	*Zenaida macroura*	0	0	0	0	0	0	1-F	0	1
Red-naped Sapsucker	*Sphyrapicus nuchalis*	0	1-F	0	0	0	0	0	0	1
Black-capped Chickadee	*Poecile atricapilla*	0	0	0	0	1-W	0	0	0	1
Townsend’s Solitaire	*Myadestes townsendi*	0	0	0	0	1-F	0	0	0	1
American Robin	*Turdus migratorius*	0	1-W	0	0	0	0	0	0	1
House Finch	*Haemorhous mexicanus*	0	0	0	1-F	0	0	0	0	1
Brewer’s Sparrow	*Spizella breweri*	0	0	0	0	1-F	0	0	0	1
Dark-eyed Junco	*Junco hyemalis*	0	0	0	0	0	1-F	0	0	1
Orange-crowned warbler	*Leiothlypis celata*	0	0	0	0	1-F	0	0	0	1
Lazuli Bunting	*Passerina amoena*	0	0	0	0	0	1-F	0	0	1
Unknown bird		0	0	1-W	0	1-F	0	0	0	2
Totals		14	11	1	1	5	5	2	0	39

**Notes.**

W winter F fall season

The addition of fall season observations yielded collisions for 11 different species, 9 of which only were observed in fall. Fall observations included several migrants such as Lazuli Bunting (*Passerina amoena*), Lincoln’s Sparrow (*Melospiza lincolnii),* and Orange -crowned Warbler (*Leiothlypis celata*).

Table 3 summarizes whether the significant risk and protective factors identified in past research replicate, when the data collection interval includes a fall migration in addition to a winter season. When entered as single predictors, each of the three were significant. The presence of pear trees increased odds of collision by 3.33, mirrored windows increased odds by 3.92, and bird-friendly glass reduced odds by 84% (odds ratio = 0.16). When the three predictors were combined, the unique variance accounted for by each factor was reduced due to correlations among the factors. However, mirrored windows were still a significant risk factor (*p* = 0.03) and bird-friendly glass was marginally non-significant (*p* = 0.09). All 1-, 2-, and 3-factor combinations were analyzed and the combination of pear trees and mirrored windows had the best fit (QICC unconditional = 241.70 and QICC best fit model = 236.74). In the best fit model, pear trees increased odds of collision by 2.31 and mirrored windows increased odds by 2.33.

**Table 3:** Fall and winter bird-window collisions per day predicted by building risk and protective features: Generalized estimating equation results for replication of Brown et al. (2019).

					95% Wald C.I.		*p*-value, original
	B	S.E.	*p*	Exp(B)	Lower	Upper	Study
Model 1: All three predictors							
Intercept	−2.80	0.30	0.01	0.06	0.03	0.11	0.01
Pear trees	0.49	0.42	0.24	1.64	0.72	3.71	0.04
Mirrored windows	0.85	0.39	0.03	2.33	1.08	5.05	0.09
Bird-friendly glass	−1.30	0.77	0.09	0.27	0.06	1.23	0.78
Model 1: All three predictors							
Intercept	−3.14	0.28	0.01	0.04	0.03	0.08	0.01
Pear trees	1.20	0.34	0.01	3.33	1.71	6.49	0.01
Model 3: Mirrored windows only							
Intercept	−2.82	0.20	0.01	0.06	0.04	0.09	0.01
Mirrored windows	1.37	0.33	0.01	3.92	2.04	7.54	0.01
Model 4: Bird-friendly glass							
Intercept	−2.28	0.16	0.01	0.10	0.07	0.14	0.01
Bird-friendly glass	−1.82	0.73	0.01	0.16	0.04	0.67	0.11
Model 5: Pear + mirrored, best fit							
Intercept	−3.14	0.28	0.01	0.04	0.03	0.08	0.01
Pear trees	.84	0.40	0.04	2.31	1.05	5.06	0.01
Mirrored windows	.85	0.39	0.03	2.33	1.08	5.05	0.09

**Notes.**

B B coefficient S.E. Standard error of B Exp(B) odds ratio CI confidence interval

Data were collected in fall 2019 and winter 2020 at the University of Utah, Salt Lake City, Utah, USA.

The addition of fall data was associated, as expected, with a significant protective role for bird-friendly glass (*p* = .01) compared to the winter-only data analyzed by Brown et al. (2019), where bird-friendly glass had been non-significant (*p* = 0.11). As shown in Table 1, buildings 4 and 5 experienced four window collisions each in fall and only one window collision each in winter. The addition of fall data meant that two buildings with bird-friendly glass had, across two seasons, lowered odds of bird collisions. There were only two total window collisions at the building with ORNILUX^®^ UV glass; as noted in the methods section, one of those might have been a carcass dropped from a building perch after a bird fed on it. Pear trees and mirrored windows continued to be significant risk factors across the two studies, as shown in the comparison of significance levels of risk factors across the current and prior data sets (see Table 3).

To assess whether frugivorous birds like Cedar Waxwings were especially vulnerable in winter because of pear trees planted near reflective or mirrored windows, we tested for a fruit tree by season interaction. The interaction term, in Table 4, was significant, with the combination of winter season and pear tree proximity yielding a 40-fold increase in odds of collision. Pairwise follow-up tests showed that window collisions were higher in winter than fall for buildings near pear trees (*p* = .03). In winter, they were also higher for buildings with pear trees than without pear trees (*p* = 0.04). The number of collisions did not differ between winter collisions near pear trees versus fall collisions away from pear trees. The addition of fall data on collisions at buildings with extensive glass exteriors on the north end of campus (Buildings 5 and 6) essentially equaled the per day collisions from the two buildings with high winter window collisions (Buildings 1 and 2), given a longer winter than fall season observation period.

**Table 4:** Interactive effects of pear tree proximity and season on bird-window collisions: Generalized estimating equation results.

					95% Wald C.I.	
	B	S.E.	*p*	Exp(B)	Lower	Upper
Intercept	−2.01	0.34	0.01	0.13	0.07	0.26
Pear trees	−1.81	0.81	0.03	0.16	0.03	0.80
Mirrored windows	0.85	0.39	0.03	2.33	1.08	5.05
Bird-friendly glass	−1.30	0.77	0.09	0.27	0.06	1.23
Season (winter =1)	−1.82	0.66	0.01	0.16	0.04	0.59
Pear trees * Season	3.69	0.99	0.01	40.00	5.78	277.05

**Notes.**

B B coefficient S.E. Standard error of B Exp(B) odds ratio CI confidence interval

Data were collected in fall 2019 and winter 2020 at the University of Utah, Salt Lake City, Utah, USA.

## Discussion

### Bird-friendly windows

We found that two buildings with bird-friendly windows had fewer collisions than other buildings when data were collected for fall and winter seasons. Although various bird-friendly glass designs are recommended in the American Bird Conservancy web site based on data from experimental tests (American Bird Conservancy, 2020), very few peer-reviewed evaluations of their performance in buildings in situ have been published. Both the ORNILUX^®^ UV protection and the fritted windows have been found to protect birds in experimental trials where the windows are tested without being installed in actual buildings (Klem, 2009; Klem & Saenger, 2013). Our study represents the first of many studies needed to evaluate how these bird-friendly windows perform in practice. As research demonstrates, buildings can have a wide variety of environmental features that influence their likelihood of window collisions, such as nearby trees, siting for shadows and light, species abundance (Nichols et al., 2018; Wittig et al., 2017), or shape of the building (Riding, O’Connell & Loss, 2019). Thus, it is important to test bird-friendly windows across a wide variety of design and habitat features. Although bird-friendly windows in building applications receive anecdotal support, such as the reduced window collisions reported by the Detroit Zoo (Van Dam, 2018), this study provides more systematic support for the efficacy of ORNILUX^®^ and fritted windows.

Two forms of bird-friendly windows were evaluated in the current study. Tunnel testing of fritted glass has suggested that frit patterns applied as stripes were more effective than dots for the same area of glass covered (Sheppard, 2019). However, the dotted frits in the present study were effective. Fritted windows had no collisions in the current study and only one collision (a feather pile) in the 2019 study. It is not clear why dotted frits are so effective in the current study. The (Sheppard, 2019) study had tested white 3.2 mm dots that were spaced to cover only 20% of the window surface. The current study tested “warm grey” 3.2 mm dots were spaced approximately five per 25 mm, with approximately 40% coverage. Another anecdotal report has indicated that earlier tests of 3.2 mm white dots spaced to cover 40% of the surface did deter collisions (Leonard, 2013). The closely spaced dots in the present study might be perceived as a solid surface, not individual dots. This suggests it would be fruitful to test differences across frit shapes, colors, and coverage.

For the bird-friendly building with ORNILUX^®^ windows, there had been no window collisions in the 2019 study and there were two window collisions in the current study. The Lincoln Sparrow fatality was a clear window collision where windows were recessed under a substantial overhang, which lowers the visibility of the UV patterns and therefore effectiveness of the protection, according to the manufacturer’s web site (http://ornilux.com). The Mourning Dove fatality might have been a carcass cleaned by a predator or scavenger, given the condition of the carcass. We note that one published study has separated intact carcasses from those with evidence of dismemberment to include in separate analyses; dismembered carcasses might result from scavenger activity after a window collision or from predation events that deposited the carcass in a manner where it is indistinguishable from the defining features of a window collision (Loss et al., 2019). Future research on larger samples might benefit from this strategy of separate analyses, with groups of carcasses defined as clear window collisions versus potential window collisions (Loss et al., 2019). In either case, by keeping the Mourning Dove in the data as a window collision, we provided a conservative test of the efficacy of the ORNILUX^®^ glass. Interest in fritted glass and ORNILUX^®^ UV glass is likely to increase as building owners grow more aware of the need to protect birds to attain more sustainable buildings (Sheppard, 2019).

### Seasonality of collisions

The current study provided insight into winter collisions, whereas past research has often focused on bird fatalities in migration or breeding seasons. Many studies find fall to be the season of most window collisions, given that fall migrations include many young birds that may not make a spring return (Zink & Eckles, 2010). Consequently, several studies have collected data during fall only (Hager et al., 2017) or fall and spring migration only (Bracey et al., 2016; Cusa, Jackson & Mesure, 2015). Sometimes the fall fatality rate is especially high; in Cleveland, Ohio there were 193 fall and 52 spring migrant collisions, although with more observation days in fall, and substantially fewer found in summer and winter (Borden et al., 2010). In Minneapolis, Minnesota there were 758 fall (2.5 months), 209 spring (2.5 months), and 33 window collisions in summer (June) (Loss et al., 2019). In Blacksburg, Virginia; Alberta, Canada (Kummer, Bayne & Machtans, 2016b), San Francisco, California (Kahle, Flannery & Dumbacher, 2016), and Rock Island and Moline Illinois (Hager et al., 2013), fall and spring migration as well as summer breeding seasons had more window collisions than winter (although the 5 winter collisions in Illinois were close in magnitude to other seasons: spring *n* = 7, summer *n* = 8, and fall *n* = 14). Not all studies share the pattern of fall prominence of window collisions. A Stillwater, Oklahoma study identified more spring than fall and summer window collisions (Riding, O’Connell & Loss, 2019) and a Xalapa, Mexico study found no seasonal variations (Gómez-Martínez et al., 2019). This short review suggests that there are likely more fatalities in migratory seasons, especially fall. However, when studies omit winter data collection, they may underestimate risks to resident birds who are present in winter.

Although many studies describe collisions at each season or analyze multiple seasons of collisions together, the current study represents one of several that has tested for particular seasonal differences in risk factors or species vulnerability. The season by pear tree interaction test provided more definitive data than past research. The pattern tentatively identified by Brown et al. (2019) was that winter proximity of fruiting pears to buildings with mirrored and reflective windows posed special risk. However, without data from another season, it was difficult to be definitive. The new data indicated that the combination of pear trees near buildings in winter posed a 40-fold increased risk of bird window collisions over other conditions. Other studies have taken different strategies to understand different signatures of risk across seasons. For example, Riding et al. conducted separate analyses by seasons and found that risk factors vary across seasons, with lawn cover, for example, important in summer and fall but not spring (Riding, O’Connell & Loss, 2019). Similarly, another study found night-lighted buildings to be more risky in spring and larger total glass area to be more risky in fall (Loss et al., 2019).

### Cedar Waxwing vulnerability

Given the research field’s emphasis on migratory season data, risks for birds like Cedar Waxwings, who are drawn to winter fruiting trees, may also be underestimated. Despite relatively few studies of winter window collisions, it is notable that the review by Loss et al. (2014) found that Cedar Waxwings were the fifth most vulnerable to window collisions, with 3.6 times the collision risk as other species (Loss et al., 2014). However, the current study and the original Brown et al. (2019) study documented a larger percentage of Cedar Waxwing window collisions than found in other studies. The Brown et al. (2019) review of prior research found that Cedar Waxwings percentages of the total collisions were 15.7% in Durham, North Carolina (Ocampo-Peñuela et al., 2016) and 14% in Edmonton, Alberta (Kummer & Bayne, 2015) but less than 3% in seven other studies. One way to assess Cedar Waxwing vulnerability is to compare the collision data to the percent of eBird lists with Cedar Waxwing sightings (although this measure does not account for the numbers of birds seen per species, and Cedar Waxwings are often in flocks). The percentage of eBird lists with Cedar Waxwings in Salt Lake City is 9.1% for November through January, much lower than the 62.2% of collisions in the current study or the 91% found in the earlier winter-only study (Brown et al., 2019). For the other two studies with the highest percentages of Cedar Waxwings, Durham had 2.9% of eBird lists with waxwings in the target months (April and September/October) compared to 15.7% of waxwings in collision data; Edmonton had 8.8% in eBird lists (year-round) compared to 14% in collision data. These comparisons suggest that both Durham and Edmonton might also have local conditions that put Cedar Waxwings at greater than expected risk. Our research showed that the very local conditions of winter food from pear trees near mirrored or reflective window buildings create high risks for Cedar Waxwings. Pear trees are becoming more abundant across the country through invasive spread and inexpensive plantings (Culley & Hardiman, 2007; Tallamy, 2009), potentially multiplying the number of sites where pear trees are near windows. We urge other researchers to be alert to the possibility that their research sites might have this deadly combination of pear trees near windows and to collect data on whether other fruiting trees are also deadly to Cedar Waxwings when planted near windows. In addition, given that Cedar Waxwings flock all year and are very frugivorous, it is not clear whether fruit trees near windows pose risks to other birds; we witnessed European Starlings (*Sturnus vulgaris*) and American Robins (*Turdus migratorius*) in the trees but recorded only one robin fatality near the pear trees.

### Targeted mitigation

As knowledge of bird window collision risk factors increases, it will be important to figure out how to apply this knowledge to enhance bird safety. For example, if winter deaths of Cedar Waxwings are found elsewhere, it might be possible to apply tempera paint to especially risky windows, washing it off after winter season (Sheppard & Phillips, 2015). Due to aesthetic concerns and challenges of reaching window surfaces, this proposed solution will not work for the buildings at the University of Utah, where we plan to mitigate the risky windows with applications of external dots (Feather Friendly^®^). Other immediate solutions could involve replacing problematic trees. For long-term bird protection, planting guidelines could forbid planting fruiting trees near expanses of glass. Bird-friendly building standards that require permanent bird-friendly glass are becoming more popular but are not yet common. In addition to recommending such building standards at state or city levels, universities could also foster bird-friendly building requirements. Mirrored windows allow humans unimpeded views of the outdoors but, based on the evidence of their deadliness, we believe they should not be used in the future where birds might be found. The expansive views from mirrored windows, if motivated by a desire to see and appreciate nature, could be satisfied by bird-friendly windows instead.

### Limitations

The study is limited by a small sample size of eight buildings and two seasons. Nevertheless, our targeted research questions could be addressed with a limited subset of conceptually- and empirically-based variables. In the future, it would be useful to collect data across larger samples that mix the risk and protective factors in various combinations and begin to single out differences among bird-friendly glass solutions. In addition, the study was consistent with a past published study, providing a rare opportunity to replicate past research, which has been called for by the bird window collision research community (Loss et al., 2019). We were also limited in that observer availability varied by time of day, although all observations were made during daylight hours. Like many studies of bird window collisions, we acknowledge that our detection of bird window collisions was unlikely to be perfect, so that the numbers should be considered underestimates.

## Conclusions

We were able to confirm our expectations of the roles of pear trees, mirrored windows, and bird- friendly glass in the current study. Fruiting pear tree proximity and mirrored windows increased risks of bird window collisions. Bird-friendly windows, specifically fritted windows and ORNILUX^®^ UV-patterned windows were associated with lower risks of bird window collisions. Pear tree proximity to buildings in winter created a 40-fold risk of bird window collisions, especially for Cedar Waxwings. We urge researchers to conduct more winter season bird window collision studies in order to test the generalizability of these effects. We also urge more attention to the combination of trees and buildings that provide special risks for birds. The use of iNaturalist to document findings can support citizen awareness, advocacy, and problem-solving for bird conservation (Chandler et al., 2017). Finally, we encourage more researchers to use the evidence from bird window collision studies to influence building and landscaping policies in the study areas.

##  Supplemental Information

10.7717/peerj.9401/supp-1 Supplemental Information 1 Raw data and variable definitions

## Additional Information and Declarations

### Data Availability

The following information was supplied regarding data availability: Raw data is available as a Supplementary File.
